# Assessing Communication during Remote Follow-Up of Users with Pacemakers in Norway: The NORDLAND Study, a Randomized Trial

**DOI:** 10.3390/ijerph17207678

**Published:** 2020-10-21

**Authors:** Daniel Catalan-Matamoros, Antonio Lopez-Villegas, Knut Tore Lappegård, Remedios Lopez-Liria

**Affiliations:** 1 Health Research Centre, University of Almeria, 04120 Almeria, Spain; dacatala@hum.uc3m.es (D.C.-M.); rll040@ual.es (R.L.-L.); 2 Department of Communication Studies, University Carlos III of Madrid, 28903 Madrid, Spain; 3 Division of Medicine, Nordland Hospital, 8005 Bodø, Norway; knut.tore.lappegard@gmail.com; 4 Institute of Clinical Medicine, Faculty of Health Sciences, University of Tromsø, 9010 Tromsø, Norway; 5 Social Involvement of Critical and Emergency Medicine, CTS-609 Research Group, Hospital de Poniente, 04700 Almeria, Spain; 6 Department of Nursing Science, Physiotherapy and Medicine, University of Almería, 04120 Almeria, Spain

**Keywords:** pacemaker follow-up, remote monitoring, hospital monitoring, healthcare communication, cardiovascular diseases

## Abstract

As effective communication is a key ingredient for the provision of quality healthcare services, this study aimed to explore the communication experiences in the remote monitoring of older adults with a pacemaker. The study was based on a non-masked randomized observational design. The Healthcare Communication Questionnaire and in-depth interviews were conducted for data collection. A total of 49 patients participated in the study. The study findings reveal overall positive communication experiences by pacemaker users in remote monitoring with no significant differences from users in hospital monitoring. The remote option is perceived as safe and convenient, and communicating with the clinicians from home is considered comfortable and confidential. The study provides insights into the content of communication experiences in telehealth and practical implications in healthcare contexts. In a world that increasingly relies on remote communication, it is crucial to match technologies to patient needs and assess communication with patients. This will ensure the success of new models of care and establish appropriate criteria for the use of telehealth services. These criteria are all relevant in the implementation of health technology in the future as a part of effective patient-centered care.

## 1. Introduction

Cardiovascular diseases (CVDs) are a leading cause of morbidity and the most common cause of mortality in Europe, leading to almost twice as many deaths as cancer [1]. Among CVDs, myocardial infarction is considered as one of the top five most prevalent specific causes of death [2]. 

Pacemakers (PMs) are common implantable medical devices (IMDs) that are surgically placed inside a patient’s body [3]. It is estimated that each year, 1.25 million permanent pacemakers are implanted worldwide, and approximately 500,000 were implanted in patients in Europe in 2016 [4]. Indications have broadened from originally only atrioventricular block to also include sinus node disease and other rhythm abnormalities, causing a continuous growth in the clinical use of PMs [5]. Pacemakers are devices that help in monitoring abnormal heart rhythms and regulate heartbeats, and they are most commonly implanted in the chest region [6,7]. According to clinical guidelines, patients with an implanted PM need to be followed up every 3–12 months [8,9]. Remote follow-up services are considered a cost-effective option because of an increasing number of patients attending cardiology services [10]. Remote healthcare services provide several advantages such as increasing system sustainability, improving demand management, reducing hospital stays, decreasing the number of health consultations and journeys, improving communication among clinicians and the better access of users to healthcare services [11,12]. 

In the Norwegian Coordination Reform for the healthcare sector [13], special attention was paid to telehealth strategies. The use of telehealth is growing in healthcare services. Initially, telemonitoring and remote follow-up were used to provide healthcare access to more remote populations [14]. New telecardiology strategies include remote monitoring (RM) to check system integrity, alerts on arrhythmic episodes, and potentially replacing in-clinic follow-ups and managing disease remotely [15]. On comparing patients with RM with those undergoing conventional follow-up in hospitals, similar frequencies of emergency visits and re-hospitalizations between the two groups were observed [16]. Additionally, the RM option is a significantly cost-effective alternative [17] and has a remarkable impact on the informal care given by the relatives and friends of patients with PMs in terms of their well-being and cost [18].

As remote healthcare services and technology are quickly becoming commonplace for healthcare organizations across the globe, it is urgent to carry out an associated in-depth study [19]. In relation to the RM of pacemakers, while many economic evaluations and outcome studies have been conducted, only a few have focused on the perception of communication by patients living with pacemakers. Therefore, the present study focused on the communication perception of people living with remotely monitored pacemakers. 

### Communication and Monitoring of Pacemakers

Effective communication is key to the provision of quality healthcare services, and communication between clinicians and patients is fundamental within the framework of “patient-centeredness” [12,20,21]. Chronic heart failure is a progressive condition characterized by an uncertain clinical trajectory, with a high risk of morbidity and mortality, that affects over 8% of older adults, globally [22]. Implantable electronic cardiac devices have revolutionized therapy in cardiology and are recommended in both national and international guidelines to treat chronic heart failure secondary to left ventricular systolic dysfunction [23]. The average age of pacemaker recipients is 75 ± 10 years [24]; therefore, communication efforts should be clearly addressed for the elderly population. In this case, the understanding and management of illness are challenged by the presence of multimorbidity, polypharmacy, and frailty. For example, in the United States, older adults with heart disease, on average, have five other chronic conditions [25] and consume an average of 11 different medications daily [26], and nearly 76% have described feeling frail [27]. Consequently, the complexity of this population group challenges health professionals, patients, and informal caregivers to engage in communication with pacemaker users.

Pacemaker implantation is a very important life event, and therefore, patients may have different and particular experiences of living with such a device. Thus, prior to remote monitoring implementation, all involved personnel, including patients, must be well informed and educated about the RM process and expectations [28]. For example, users should be informed that they are responsible for keeping their contact information updated, informing about extended travel, maintaining the function of the transceiver and appropriate landline/cellular communications, and showing up for in-person assessments when an alert is generated and when asked by the health professionals. 

Older adults with heart disease and other chronic conditions require frequent communication considering their declining conditions [29]. These conversations occur infrequently between patients and caregivers [30]. Besides, there is a lack of patient-friendly information about telehealth systems, and patients often rely on third-party information (e.g., on the Internet), which can be inaccurate or even wrong [3]. Patient-related barriers are often considered the most important; hence, patients’ perspectives can yield valuable insights for informing clinical approaches for the effective integration of these new remotely monitored pacemakers. 

Earlier research on the elderly population, chronic heart disease, and communication focused mostly on end-of-life communication and the delivery of bad news without including analyses about other topics [29,31,32]. To our knowledge, no previous research exists regarding the perceptions of PM patients about communication during remote monitoring. Increasing our knowledge about the experience of users’ communication during the remote monitoring of pacemakers could identify important opportunities for improvement. The perceptions of engaging in communication from the perspective of older adults with remotely monitored pacemakers remain poorly understood from the research perspective. To address this gap, this study explored the communication experiences in older adults with heart conditions, specifically, PM users following remote monitoring. The rationale of this study was to produce pertinent and translatable knowledge for future opportunities in the context of the healthcare of these patients and to direct future research.

## 2. Materials and Methods 

This study is a part of a larger project, the NORDLAND study, which has conducting specific economic evaluations and assessing health-related quality of life, users’ experiences, and the communication of these new telehealth services since 2014. This project entails collaboration among chronic heart patients with a pacemaker, their relatives, cardiologists, nurses, psychologists, and health communication experts.

This study follows a non-masked randomized observational design. The participants, having been implanted with a pacemaker, were required to be followed up either in the hospital or remotely. The recruitment took place in Nordland Hospital, Bodø, Norway. This hospital hosts a pacemaker center that covers 170,000 inhabitants and offers around 80–90 PM implants per year.

The study protocol was as per an earlier study [33]. Every participant had been scheduled for a pacemaker implant. The inclusion criteria were an age of 18 years or older, the capacity to provide informed consent and to maneuver the home monitoring system, and a life expectancy > 1 year. The exclusion criteria were being scheduled for an implantable cardioverter–defibrillator (ICD) or cardiac resynchronization therapy (CRT), and simultaneous involvement in other studies. All patients were included consecutively in the study and were followed up for 12 months after the PM implants. Seventy-six patients were identified in the hospital database, and 50 were invited to participate and randomized to either remote monitoring (RM, n = 25) or hospital monitoring (HM, n = 25), before being implanted with the PM. The investigators were not aware of or did not influence the randomization result prior to inclusion. After this, and due to the explicit nature of the intervention, it was not possible to blind patients or clinicians to the identity of the group to which they had been randomized.

The patients were implanted with either a single (VVIR) or a dual chamber (DDDR) pacemaker based on their diagnosis. Further characteristics and descriptions of the devices that were received by each group of participants are mentioned in detail in a previously published article [16,33]. 

Data were collected at 6 months and 12 months after surgery. At 6 months, 25 RM patients and 24 HM patients participated, with each participant answering a set of questions. One HM patient could not attend due to unavailability. The participants answered an adapted version of the HCCQ (Health Care Communication Questionnaire) [21], which is listed in Table 1. The HCCQ aims to measure outpatients’ communication experiences with hospital healthcare professionals. To further investigate the communication experiences of the RM participants 12 months after the implants, the 25 participants from the RM group were invited to attend an in-depth interview. In the total sample, four patients (RM: 2; HM: 2) died from non-cardiovascular causes. In addition, in the RM group, 11 were unavailable to attend the interview session. Therefore, 12 in-depth interviews were conducted. A member of the research team was in charge of conducting these interviews by using an interview guide including open and closed questions (Table 2). The interview guide was designed to elicit participants’ communication experiences in relation to the RM of her/his pacemaker.

The above protocol was approved by the Regional Ethics Committee (REK Nord; with the reference number: 2014/383/REK Nord). The study was developed according to the tenets of the Declaration of Helsinki. All patients signed corresponding informed consent forms before their enrollment (patient recruitment and follow-up, from 31 August 2014 to 30 November 2016), and appropriate measures were taken to ensure data privacy. The trial protocol was registered at ClinicalTrials.gov, Identifier: NCT02237404. 

The statistical analyses were carried out following an earlier published study [18]. To start with, patient baseline characteristics and potential differences between groups were compared using a difference-in-means test for continuous variables and a difference-in-proportions test (binomial method) or the chi-square test (replaced by Fisher’s exact test for cells with n < 5 cases) for qualitative variables. Next, the results from the questionnaire were presented on a single question based on a comparison between the two groups, telemonitoring and hospital monitoring, using the Mann–Whitney U test for ordinal data and the chi-square test for nominal data. The analysis of the in-depth interviews followed a framework approach, whereby a coding frame was inductively constructed from the data [34,35]. Analyses were carried out using the SPSS statistical software 24th edition (SPSS Institute, Inc., Chicago, IL, USA) and NVivo 11th edition for content analysis.

## 3. Results

The baseline characteristics are presented in Table 3. General information from the six-month follow-up after the implant is listed in Table 4. There was no significant difference between the RM and the HM groups with regard to age, gender, pacing indication, and other clinical characteristics. Differences were only observed in those items that were directly influenced by the type of monitoring, including “transmissions from patient’s home” and “calls/letters sent to the patients”. 

At 6-month follow-up, 24 participants from each group answered the questionnaire about the results derived from the HCCQ. The overall communication experience with both types of follow-ups was positive, and there was no significant difference between the remote monitoring and the hospital monitoring groups (see Table 5).

After 12 months of surgery, the participants from the RM group were invited for an in-depth interview. In total, 12 participants accepted and attended the interview session, while the remaining patients declined to participate in the interviews due to unavailability or death. Each interview lasted approximately 30 min. The participants provided highly productive information that was processed. Quotes from the participants are also provided (see Table 6). In general, the participants reported positive experiences about the RM option. Considering the RM advantages, 66% of the participants (n = 8) perceived this new system as a safe option, as they felt they were being continuously followed up. Particularly, an additional advantage was reduced visits to the hospital, mainly because of the traveling time and costs. The participants also indicated positive experiences about talking to the clinicians from home, importantly, that it was easy and that they felt safe. No participant showed any dislike of these remote conversations with clinicians. The majority of the participants (n = 9.75%) did not prefer to meet the clinicians in person. Two patients (16%) mentioned that they would have preferred to meet the clinicians in person only if their health status decreased or some problems with the PM occurred. One participant (8%) mentioned his preference to meet the clinicians sometimes in the hospital for specific questions. All participants felt comfortable about talking to the clinicians from home, and also felt that the RM system was confidential. One patient (8%) mentioned having problems when conducting data transmission from home. Finally, considering what system they would prefer based on previous experience, 58% of the participants (n = 7) preferred the remote follow-up, 33% participants (n = 4) did not show any particular preference for either RM or HM, and 8% participants (n = 1) would have preferred a hospital follow-up.

## 4. Discussion

This study explored the communication experiences in PM users through remote monitoring to produce pertinent and translatable knowledge for future opportunities in healthcare contexts. To our knowledge, this is the first study to explore the follow-up communication experiences of PM users living remotely. Our findings revealed (a) overall positive communication experiences in RM pacemaker users, (b) no differences in the communication experiences between the RM and HM users, (c) the perception of remote follow-up as a safe and confidential option, and (d) talking to the clinicians from home is a comfortable option.

Although the patients following remote or in-home follow-ups of pacemaker implants have fewer scheduled visits to the hospital and consequently less in-office contact with clinicians, our results reveal an overall positive communication experience. These patients had a scheduled visit to the hospital after one month of surgery, during which the physician explained the characteristics of the monitoring that they had been assigned to. Following the international consensus on the monitoring of cardiovascular implantable electronic devices [36], the RM patients were advised in the beginning that they would be called only if something went wrong. The RM pacemaker automatically collects and transmits encrypted health information to the Home Monitoring Service Centre, depending on the patients’ needs as defined by their physician. Therefore, after one month, no visits were scheduled in the RM group. These patients were called and referred for an in-office visit only if the received data detected any device dysfunction or a cardiovascular event.

An important outcome of our study was that the patients with in-home follow-up perceived similar communication experiences with hospital healthcare professionals to the patients with hospital follow-up. This is an important finding that is supported by a previous study that compared the two types of follow-up. However, while in this study, there was no perceived loss of communication, the earlier study was conducted in another health setting [14]. Different studies suggest that telehealth providers require high-level communication skills, for example, to compensate for a lack of visual cues [37,38,39]. Therefore, we may assume that communication by clinicians met the patients’ needs in our study. Varma (2016) states that effective communication with patients in remote follow-up should include a good presentation of the benefits of this healthcare model. Information on the expected reaction times should be cautiously described to patients, who should also be educated, along with their caregivers, on how to proceed in emergencies. Furthermore, to maintain effective ongoing communication with patients, personal knowledge of the clinicians who phone the patient when there is trouble may significantly strengthen their relationship.

Issues of patient safety in telehealth have been largely unexplored [40]. During the in-depth interviews conducted with the participants under RM, the majority of the patients perceived this type of follow-up as a safe, comfortable option and that they would prefer this remote option to the hospital follow-up. Our findings are well aligned with those of Donelan et al. [14], who observed that patients under remote follow-up perceived considerable added convenience and saved travel time, and a majority also recommended it to family and friends. Nevertheless, other authors analyzing this type of communication suggested the need for the personal contact of these patients with clinicians because patients with an insertable cardiac monitor experienced the feeling of “not knowing” or “being uninformed” [41]. In this regard, Kirkegaard et al. [42] argued that telehealth solutions cannot be a stand-alone option with no presence of the healthcare provider, especially in patients with long-term conditions. In telehealth services, the absence of visual stimuli provides unique barriers to communication and increases the risk of misunderstandings and distractions [43]. However, in the case of the remote follow-up of patients with pacemakers, there is no need for information exchange unless something goes wrong, although, in this case, personal contact was made. One month after surgery, patients are advised that in the RM environment, “no news is good news”. Our study may have found positive communication experiences because personal contact was unnecessary during the follow-up process and the remote communication experiences were perceived as successful. The sample of patients with an RM pacemaker considered this type of follow-up as safe and comfortable. However, this should be further analyzed in other health settings. For example, in a study on telerehabilitation settings including patients with heart disease, lymphedema, and chronic pulmonary obstructive disease, personal contact with clinicians was perceived safer by patients [44]. Furthermore, some other studies recommend that as these patients are often suffering mentally from depression or anxiety, clinicians should be aware of significant mental health symptoms that cannot be perceived by the remote follow-up system [45,46].

Therefore, the authors suggest a balanced remote follow-up system that includes several phone calls, even when the system has not declared any unusual event. Considering that this group of patients is specially aged with high comorbidity, there may be queries related to the pacemaker, especially when they are asymptomatic; thus, this contact is the only mode for knowing about their health status and confirming their progress. This induces us to reflect on the following Albert Einstein quote: “The human spirit must prevail over technology”.

We recognize limitations to this work, which need to be considered before making any interpretation of the results. Measuring a patient’s communication experience includes feedback biases from intentionally answering the questionnaires to accomplish positive outcomes. Our overall positive communication experiences could have been influenced by the phenomenon called the “Hawthorne effect” [47], which states that patients tend to change their behavior when they are targets of interest and attention, irrespective of the specific nature of an intervention, which could be a limitation of our results. In such situations, patients may become eager to please their clinicians and make them feel successful. In addition, some patients wish to participate so that “good” results can be achieved in the study. Particularly, most data describing communication experiences come from retrospective approaches using questionnaires, interviews, and focus groups. Such retrospective data are subject to recall bias. Further research must be carried out using more reliable techniques such as observation and/or recording to assess skills in communication between clinicians and patients in real time [48]. Another limitation is that we conducted an open study wherein the clinicians, researchers, and patients knew the follow-up types. Finally, we would like to mention that the sample included in the NORDLAND study was small and that further research should consider strengthening this aspect. In addition, further research could also focus on analyzing the level of health and technology literacy of pacemaker users, as this could be an important point to consider in the implementation of this new technology. Nonetheless, we believe that this study presents some significantly strong points because the NORDLAND study is a randomized study in a field where it is not common to conduct such a method design. This ensures a major evidence level, a reduced chance of bias due to the random selection of the groups, and maybe repeatability and comparability with other studies. In a world that increasingly relies on remote communication, it will be crucial to match technologies to patient needs and assess communication with patients [49]. This will ensure the success of new models of care and establish appropriate criteria for the use of telehealth services. These criteria are all relevant in the implementation of health technology in the future as a part of effective patient-centered care. Telehealth should aim to improve the quality of health services with a special focus on overcoming standing barriers to healthcare when attending traditional hospital visits.

## 5. Conclusions

With the unstoppable implementation of digital contributions to meet the different needs of the patients, telehealth is expected to be adopted progressively. This study provides some insight into the content of communication experiences in telehealth and confirms positive communication experiences in cases of people with pacemakers in remote follow-up. These new health technologies might be a great complement, especially for the long-term sick population. It is thus critical to continue to develop strategies to assess and improve the communication experiences of patients. In our study, the patients reported communication experiences in a specific health setting, that of the remote monitoring of pacemakers. This study design may also be considered for other settings, as there is a lack of analysis of communication experiences with other telehealth services.

## Figures and Tables

**Table 1:** The adapted version of the HCCQ—Health Care Communication Questionnaire [21].

I was asked questions in an aggressive manner
I have been given answers in an aggressive manner
I have been treated with kindness
I have been treated in a rude and hasty manner
The healthcare provider addressed me with a smile
The healthcare provider was able to manage the consultation
The healthcare provider showed respect for my privacy

**Table 2:** Interview guide.

Why did you find being home-monitored an advantage?
What did you like about talking to the clinicians from home?
What did you dislike about talking to the clinicians from home?
Would you have preferred to have seen the clinicians in person? Why? Why not?
Did you feel comfortable talking to the clinicians from home? Why? Why not?
Did you feel that your session was confidential? Why? Why not?
Have you ever had problems in conducting any data transmission from home? Yes/No
How many times did the doctor call you to the hospital due to findings from data transmission? Number: _______
After your experience, what kind of monitoring/follow-up would you prefer?
Remote Hospital It does not matter

**Table 3:** Selected patient baseline characteristics by intervention status.

Variables	All (n = 50)	Groups		*p*-Value
		Telemonitoring	Hospital Monitoring	
Age	74.84 (±11.75)	73.68 (±14.22)	76.00 (±8.77)	0.676
Men	26 (52.0)	13 (52.0)	13 (52.0)	1.00
**Pacing indication N (%)**				
Sick sinus syndrome	24 (48.0)	12 (48.0)	12 (48.0)	0.648
Atrioventricular block	20 (40.0)	11 (44.0)	9 (36.0)	
Chronic AF with bradycardia	6 (12.0)	2 (8.0)	4 (16.0)	
**Disease manifestations N (%)**				
Syncope	14 (28.0)	8 (32.0)	6 (24.0)	0.812
Dizziness	25 (50.0)	12 (48.0)	13 (52.0)	
Dyspnea	11 (22.0)	5 (20.0)	6 (24.0)	
**Service derived N (%)**				
Emergency dept.	3 (6.0)	1 (4.0)	2 (8.0)	0.505
Cardiology ward	14 (28.0)	5 (20.0)	9 (36.0)	
Primary healthcare	4 (8.0)	2 (8.0)	2 (8.0)	
Other hospitals	29 (58.0)	17 (68.0)	12 (48.0)	
**Stimulation N (%)**				
DDDR	44 (88.0)	23 (92.0)	21 (84.0)	0.334
VVIR	6 (12.0)	2 (8.0)	4 (16.0)	
**Comorbidities N (%)**				
Dyslipidemia	27 (54.0)	13 (52.0)	14 (56.0)	0.500
Obesity (BMI > 30)	1 (2.0)	0 (0.0)	1 (4.0)	0.500
Tachyarrhythmia	18 (36.0)	7 (28.0)	11 (44.0)	0.189
Hypertension	32 (64.0)	17 (68.0)	15 (60.0)	0.384
**Other comorbidities N (%)**				
None	18 (36.0)	11 (44.0)	7 (28.0)	0.388
Others	10 (20.0)	6 (24.0)	4 (16.0)	
Coronary heart diseases	22 (44.0)	8 (32.0)	14 (56.0)	
**Pharmaceutical treatment** N (%)				
Antiaggregants	18 (36.0)	8 (32.0)	10 (40.0)	0.384
Anticoagulants	25 (50.0)	10 (40.0)	15 (60.0)	0.129
Antiarrhythmics	18 (36.0)	7 (28.0)	11 (44.0)	0.189
Antihypertensives	32 (64.0)	18 (72.0)	14 (56.0)	0.189

n = 50 (Remote monitoring group: 25; Hospital monitoring group: 25). Values are expressed as means or proportions. DDDR: Bicameral pacemaker with two electrodes placed in the atrium and in the ventricle; VVIR: Unicameral pacemaker with an electrode in the ventricle with the ability to modulate the frequency of stimulation; BMI: Body mass index. Note: this table has been previously published in a previous article [33].

**Table 4:** Follow-up general information at six months.

Variables	All (n = 49)	Groups		*p*-Value
		Telemonitoring	Hospital Monitoring	
	Number of transmissions from hospital N (%)			
0	0 (0.0)	0 (0.0)	0 (0.0)	0.26
1	41 (83.7)	21 (84.0)	20 (83.3)	
2	6 (12.2)	2 (8.0)	4 (16.7)	
3	2 (4.1)	2 (8.0)	0 (0.0)	
	Number of transmissions from patient’s home N (%)			
0	29 (59.2)	5 (20.0)	24 (100)	<0.001
3–5	15 (30.6)	15 (60.0)	0 (0.0)	
6–8	5 (10.2)	5 (20.0)	0 (0.0)	
	Extra transmissions from patient’s home N (%)			
0	45 (91.8)	21 (84.0)	24 (100)	0.12
1	1 (2.0)	1 (4.0)	0 (0.0)	
3	3 (6.2)	3 (12.0)	0 (0.0)	
	Cardiovascular events N (%)			
None	46 (93.9)	23 (92.0)	23 (95.8)	0.40
PCI	1 (2.0)	1 (4.0)	0 (0.0)	
Angina	1 (2.0)	0 (0.0)	1 (4.2)	
Lead dislodgement	1 (2.0)	1 (2.0)	0 (0.0)	
	Calls/letters sent to the patients N (%)			
0	27 (55.1)	4 (16.0)	23 (95.8)	<0.001
1	21 (42.9)	20 (80.0)	1 (4.2)	
3	1 (2.0)	1 (4.0)	0 (0.0)	
	Changes in medication N (%)			
0	33 (67.3)	17 (68.0)	16 (66.7)	0.11
1	7 (14.3)	5 (20.0)	2 (8.3)	
2	3 (6.1)	1 (4.0)	2 (8.3)	
3	4 (8.2)	0 (0.0)	4 (16.7)	
4	2 (4.1)	2 (8.0)	0 (0.0)	
	Changes in pacemaker’s programming N (%)			
0	34 (69.4)	16 (64.0)	18 (75.0)	0.34
1	13 (26.5)	7 (28.0)	6 (25.0)	
2	2 (4.1)	2 (8.0)	0 (0.0)	
	Number of hospitalizations (related or not to pacemaker’s implant) N (%)			
0	30 (61.2)	14 (56.0)	16 (66.7)	0.55
1	14 (28.6)	7 (28.0)	7 (29.2)	
2	4 (8.2)	3 (12.0)	1 (4.2)	
5	1 (2.0)	1 (2.0)	0 (0.0)	
	Number of hospitalization days (related or not to pacemaker’s implant) N (%)			
0	30 (61.2)	14 (56.0)	16 (66.7)	0.54
1–5	12 (24.5)	6 (24.0)	6 (25.1)	
6–10	4 (8.1)	2 (8.0)	2 (8.4)	
+10	3 (6.0)	3 (12.0)	0 (0.0)	
	Reasons for hospitalization N (%)			
None	30 (61.2)	14 (56.0)	16 (66.7)	0.37
Others	6 (12.3)	3 (12.0)	3 (12.5)	
Cancer	1 (2.0)	1 (4.0)	0 (0.0)	
Coronary problems	9 (18.4)	4 (16.0)	5 (20.8)	
Pacemaker dysfunction	3 (6.1)	3 (12.0)	0 (0.0)	

n = 50 (Remote monitoring group: 25; Hospital monitoring group: 24). Values are expressed as means or proportions. PCI: Percutaneous coronary intervention. Note: this table has been published in a previous article [33].

**Table 5:** Results derived from the adapted version of the Health Care Communication Questionnaire (HCCQ).

Questions	Answering Categories	Remote Monitoring Group * (n = 24)	Hospital Monitoring Group * (n = 24)	*p*-Value
Question 1	1 = Not at all; 2 = To a small extent; 3 = To a moderate extent; 4 = To a large extent; 5 = To a very large extent	1 (1, 5)	1 (1, 1)	0.383
Question 2		1 (1, 2)	1 (1, 1)	0.332
Question 3		5 (2, 5)	5 (3, 5)	0.363
Question 4		1 (1, 3)	1 (1, 1)	0.332
Question 5		4 (2, 5)	5 (3, 5)	0.431
Question 6		5 (2, 5)	5 (3, 5)	0.718
Question 7		5 (1, 5)	5 (3, 5)	0.379

* Data presented as median (min., max.).

**Table 6:** Quotes from participants in the remote monitoring group, 12 months after surgery.

“I felt safe with this continuous follow-up”, participant number 1.
“I feel that the session was confidential, I trust in the system”, participant number 16.
“It was comfortable to speak with the clinicians from home because I got the answers I needed”, participant number 22.
“Talking to the clinicians from home was safe”, participant number 34.
“One of the main advantages for the remote monitoring is that I do not have to travel to the hospital”, participant number 47.

## Data Availability

The datasets used and/or analyzed during the current study are available from the corresponding author on reasonable request.
